# Supplementary material for: Cancer cells dying from ferroptosis impede dendritic cell-mediated anti-tumor immunity
Source: Nat Commun. 2022 Jun 27;13:3676. doi: 10.1038/s41467-022-31218-2 (PMC9237053; doi:10.1038/s41467-022-31218-2)
Supplement: Supplementary file 1 — Supplementary Information [file 41467_2022_31218_MOESM1_ESM.pdf]

SUPPLEMENTARY FILE

Cancer cells dying from ferroptosis impede dendritic cell-mediated anti-tumor immunity

Bartosz Wiernicki[1,2,3], Sophia Maschalidi[2,4*], Jonathan Pinney[5], Sandy Adjemian[1,2,3], Tom Vanden Berghe[1,2,3,5], Kodi S. Ravichandran[1,2,4,6], Peter Vandenabeele[1,2,3,7,*,♯]

Affiliations:

[1]VIB-UGent Center for Inflammation Research, Ghent, Belgium

[2]Department of Biomedical Molecular Biology, Ghent University, Ghent, Belgium

[3]Cancer Research Institute Ghent (CRIG), Ghent University, Ghent, Belgium

[4] Department of Microbiology, Immunology, and Cancer Biology, University of Virginia, Charlottesville, VA, USA[5]Pathophysiology lab, Department of Biomedical Sciences, University of Antwerp, Antwerp, Belgium

[6]Division of Immunobiology, Department of Pathology and Immunology, Washington University School of Medicine, St. Louis, MO, USA.

[7]Methusalem program, Ghent University, Belgium

* Share senior authorship

♯ Corresponding author. Email: Peter.Vandenabeele@irc.vib-ugent.be

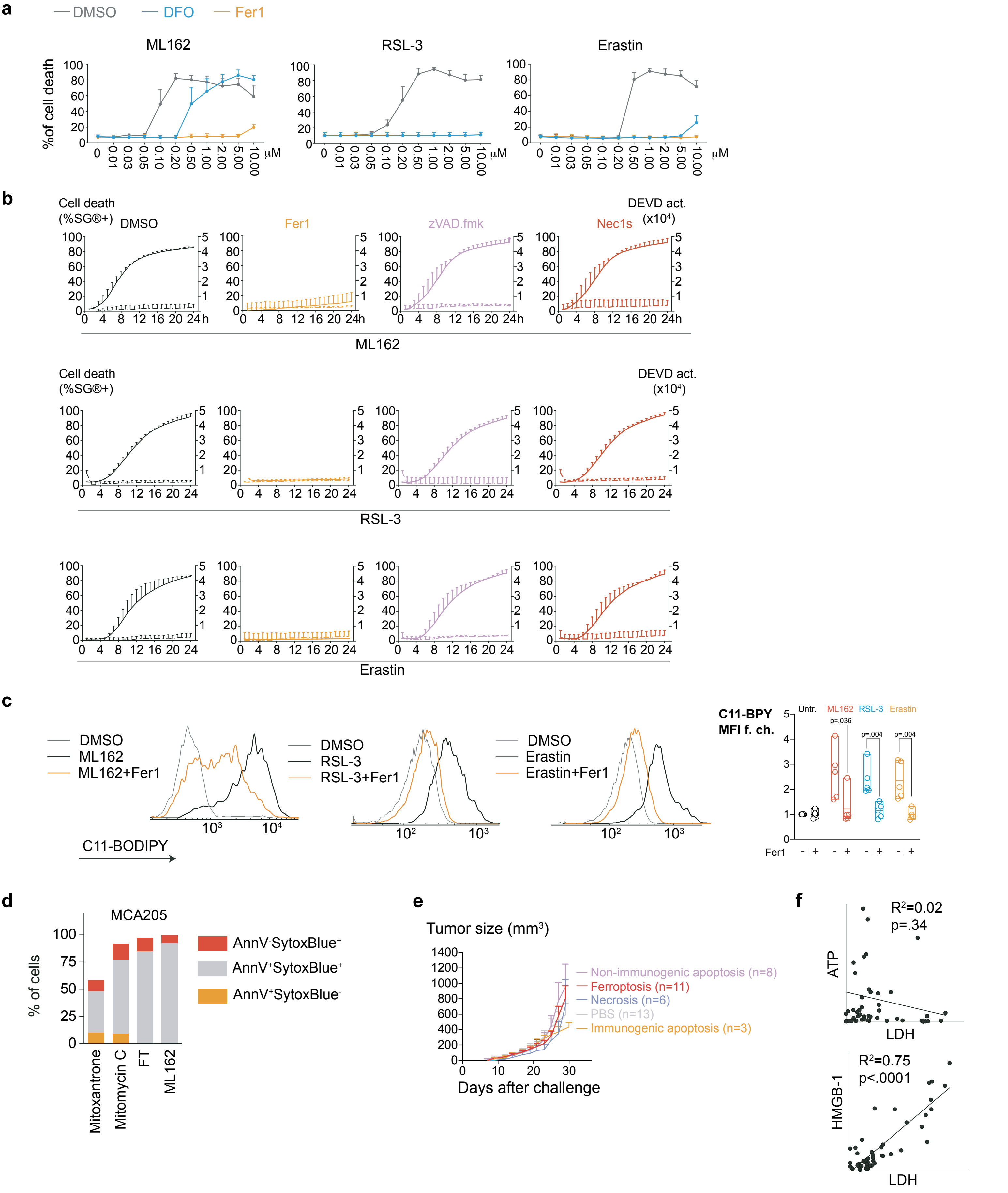

Supplementary Figure 1. Mouse fibrosarcoma cells release DAMPs during ferroptosis initiated by class I and class II ferroptosis inducers.

a. Concentration curve of ML162, RSL-3 and Erastin induced cell death in MCA205 cells. Iron chelator deferoxamine (DFO) and lipid peroxidation inhibitor Ferrostatin-1 (Fer1) were used as inhibitors. Mean ± SEM of n=3 independent experiments is presented. **b**. Unlike ferroptosis inhibitor Fer1 (0.5 μM), inhibitors of apoptosis (zVAD.fmk, 10 μM) and necroptosis (Nec1s 10 μM) do not inhibit ML162 (0.5 μM), RSL-3 (0.5 μM) and Erastin (2.0 μM) induced cell death. Mean ± SEM of n=3 independent experiments is presented. Dotted lines represent the activity of caspases. **c.** Lipid ROS levels assessed by the measurements of C11-BODIPY in MCA205 cells 2 h post ML162 (0.5 μM) or RSL-3 (0.5 μM) treatment or 8 h post Erastin treatment (2.0 μM). Fer1 (0.5 μM) was used as lipid peroxidation inhibitor. Representative of five independent experiments is shown in the form of histograms and floating bar graph shows the range (limits of the box) and median (center of the plot) of the data of n=5 independent experiments. Data was analyzed by two-sided t-test for each ferroptosis treatment with and without Fer1 administration.
d. Cell death of MCA205 cells injected as vaccines in the prophylactic vaccination model. Presented data comes from n=2 independent measurements prior to the vaccination of the animals. **e**. The analysis of the tumor growth on the challenge site in prophylactic vaccination model. Only data from animals that developed the tumor is presented as mean ± SEM. **f**. Linear regression test assessing the correlation between cell membrane permeabilization and release of ATP and HMGB1. Simple linear regression test was applied to calculate p-value and R^2.

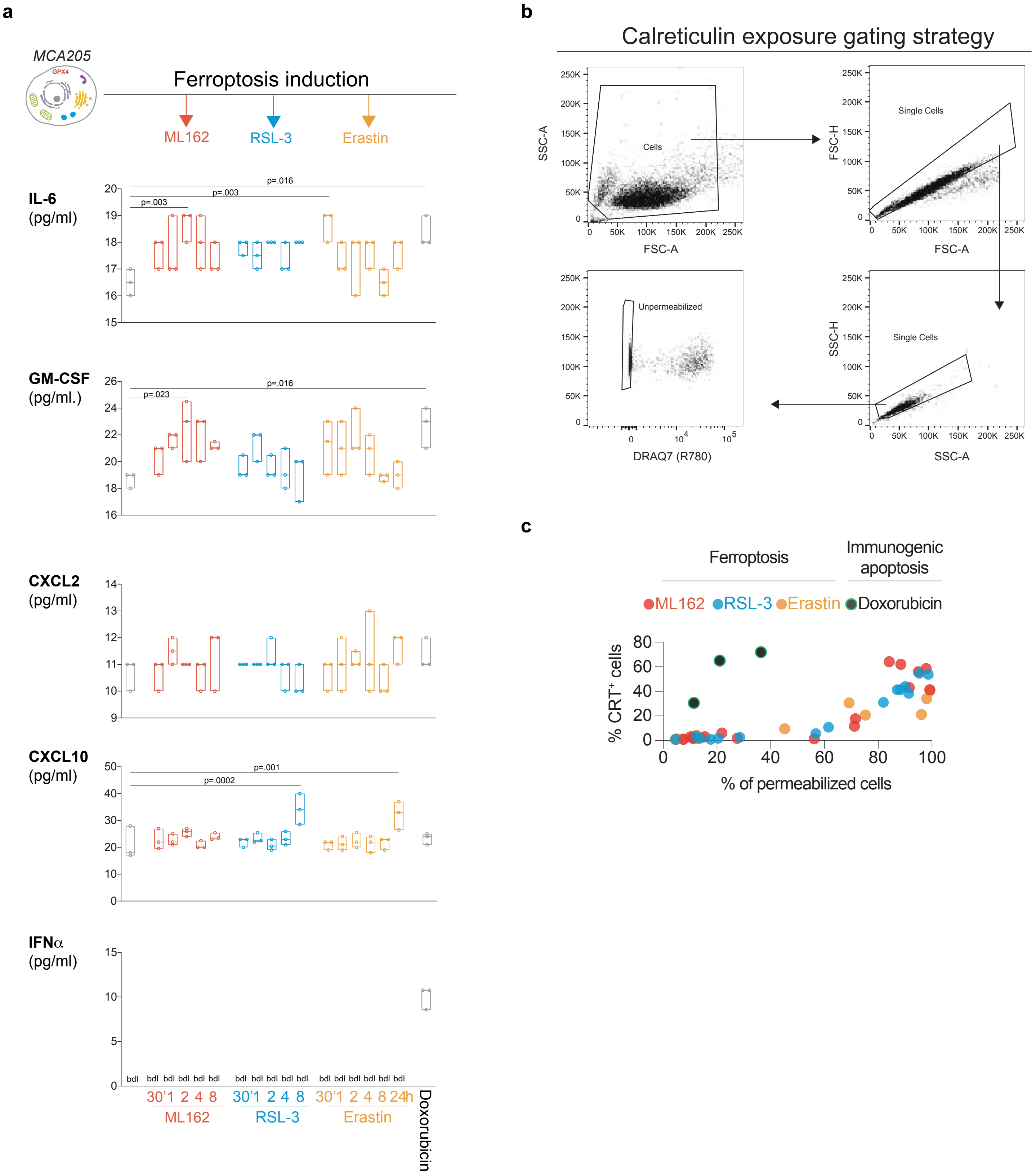

Supplementary Figure 2. The cytokine release and calreticulin exposure in MCA205 cells dying by class I and class II ferroptosis inducers.

a. The release of cytokines IL-6, GM-CSF, CXCL2, CXCL10 and IFNα after the treatment of ML162 (0.5 μM), RSL-3 (0.5 μM) and Erastin (2.0 μM) in MCA205 at different stages of cell death. Stimulation for 24 h with doxorubicin was used as a positive control. Supernatant from n=3 independent experiments was used. Data presented as floating bar with bounds representing the range of the measurements and center showing the median of observed values. One-way ANOVA with Dunnet's post-hoc test. **b**. Gating strategy for calreticulin exposure experiments. **c.** The % of MCA205 cells exposing calreticulin in the function of cell membrane permeabilization (values from Fig. 1 d).

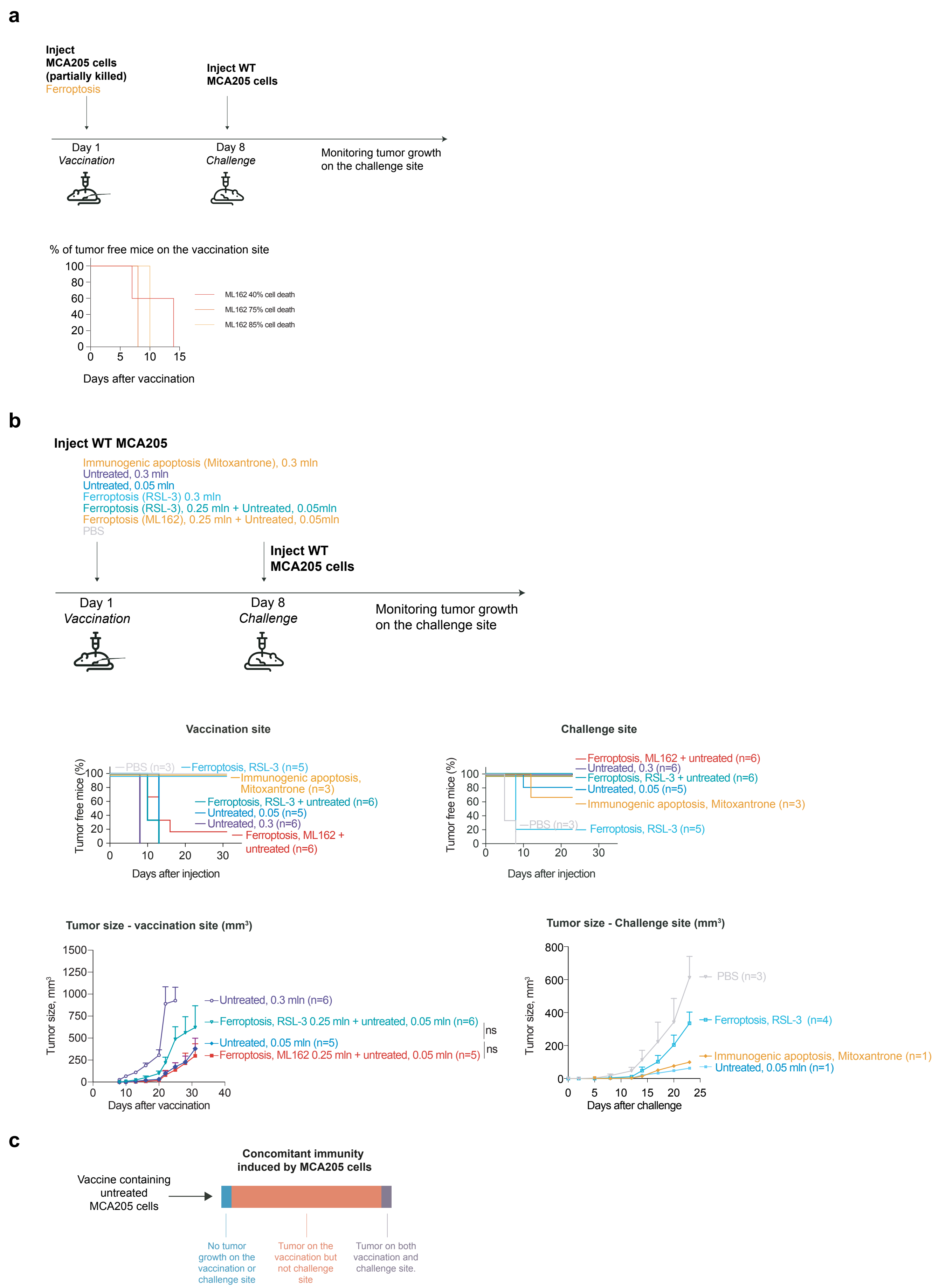

Supplementary Figure 3. Partial ferroptosis of MCA205 cells in prophylactic vaccination model causes tumor onset on the vaccination site.

a. The graph representing the percentage of tumor-bearing mice after vaccination with MCA205 cells with different levels of cell death. **b**. The analysis of concomitant immunity of MCA205 cells. Immunocompetent mice were injected with untreated MCA205 cells, untreated MCA205 cells in mixture with ML162 (0.5 µM, 14h) or RSL-3 (0.5 µM, 14h) – induced ferroptotic cells or with RSL-3 induced ferroptotic cells alone, and challenged 7 days later with the injection of live cells. Data presents the incidents of the tumor onset on vaccination and challenge site as well as the rate of the tumor growth. For the tumor size, data presented as mean ± SEM. **c.** The analysis of concomitant immunity of MCA205 cells. The graph represents the % of animals vaccinated with 0.05 x 10^6 live cells (either alone or in mix with ferroptotic cells, n=17) and subjected to the challenge with live cells, that did not develop any tumor (1 in 17 mice, blue), developed tumor only at the vaccination site (15 in 17 mice, pink/orange) and developed tumor at both vaccination and challenge site (1 in 17 mice, purple).

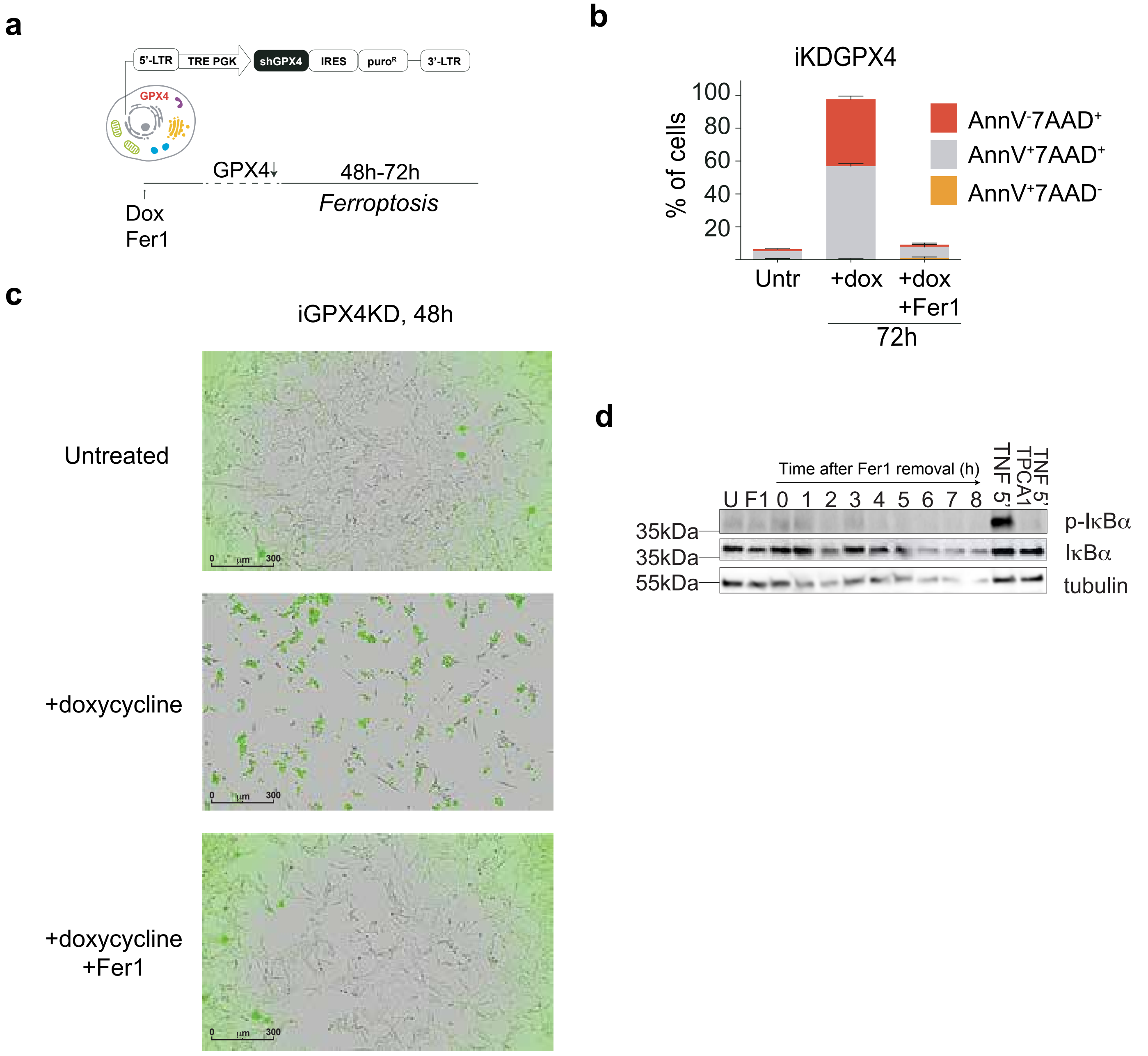

Supplementary Figure 4. Induction of GPX4 knock down in MCA205 cells causes cell death that is rescued by Fer1.
a. The scheme of cell death induction in iGPX4KD cells. **b**. Flow cytometry analysis of iGPX4KD cell death after doxycycline administration. Data presented as mean±SEM from n=3 independent experiments. **c.** Representative images of two independent experiments showing cell death in iGPX4KD 48 h after doxycycline administration. Top image represents untreated cells, while the bottom image shows rescue by Fer1. **d**. The analysis of NF-κB activation during ferroptosis. Protein from dying iGPX4KD was collected and used for western blot analysis. Untreated cells stimulated with TNF (5 min) or TNF and TPCA-1 (IKK-2 inhibitor) served as a positive and negative control respectively. One of n=2 independent experiments is presented.

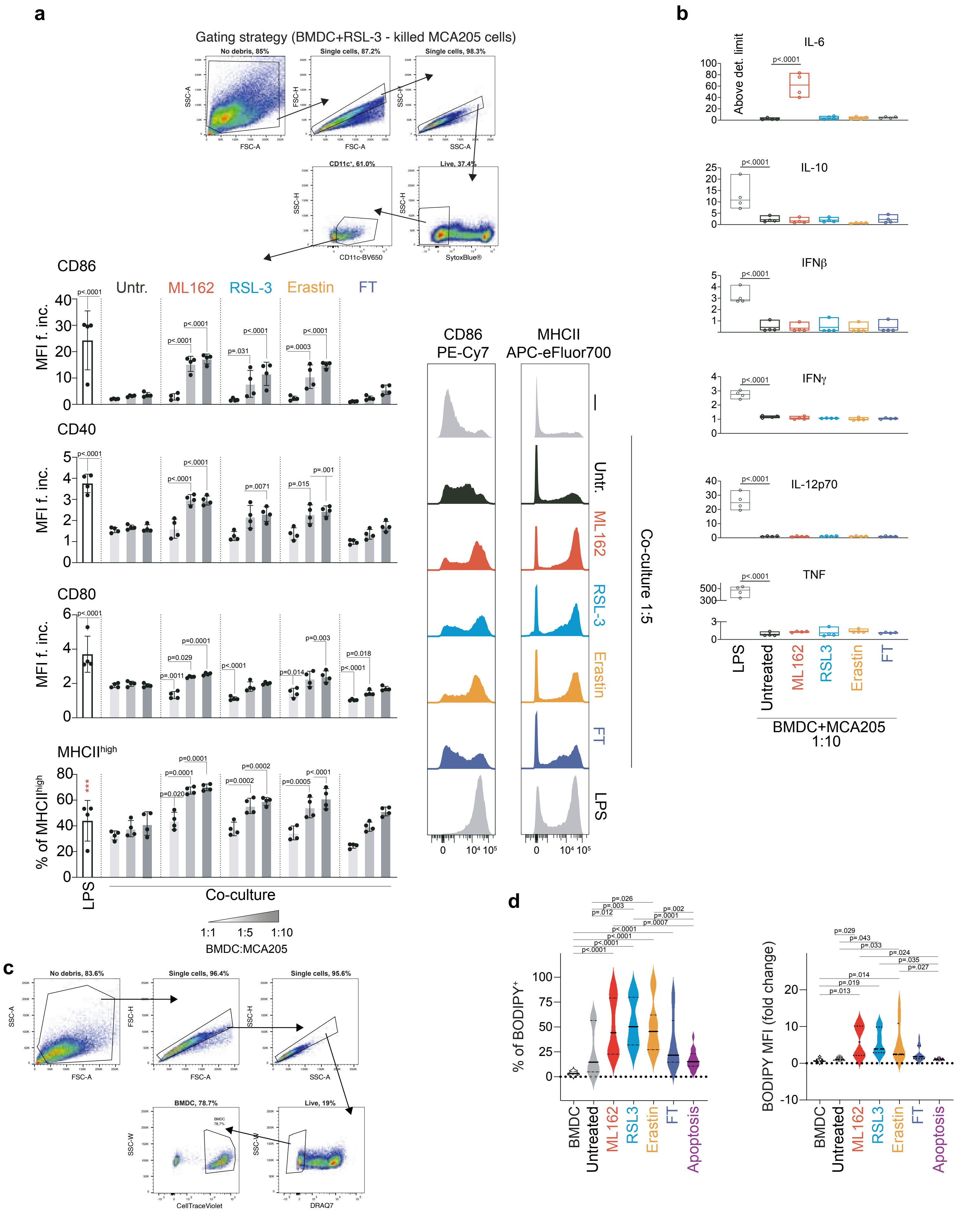

Supplementary Figure 5. Ferroptotic cells induce maturation of BMDCs, but fail to induce cytokine production

a. Maturation of bone marrow-derived dendritic cells (BMDC) incubated with untreated, ferroptotic or necrotic cells in different ratios. Data presented as mean ± SEM of n=4 biologically independent samples. Two-way ANOVA with Dunnet's post-hoc test, comparison to the BMDC control (co-culture with untreated MCA205 cells in the same ratio). Representative histograms show intensity of fluorescent signal for CD86 and MHCII after co-culture as well as after LPS treatment serving as a positive control. **b**. Cytokine release from bone marrow derived dendritic cells after the incubation with untreated, ferroptotic or necrotic cells (ratio BMDC:MCA205 1:10) or stimulated with LPS. Data presented a floating bar of n=4 biologically independent values normalized to the untreated condition. Border limits represent the range, and the center represents median of the observed values. One-way ANOVA with Dunnet's post-hoc test, p values show the comparison to the BMDC control (unstimulated). **c.** Gating strategy for analysing bone marrow derived dendritic cells (BMDC). **d**. The analysis of lipid droplets accumulation in BMDC co-cultured with MCA205 untreated, ferroptotic (ML162 0.5 μM, 14h; RSL-3, 0.5 μM, 14h; Erastin 2.0 μM, 24h), apoptotic (TNF+TAK1i, 8h) and necrotic cells (three cycles of freeze/thaw, FT). Data comes from n=10-14 independent measurements and is presented as a violin plot with median and upper and lower quartiles, One-way ANOVA with Tukey's post-hoc test.

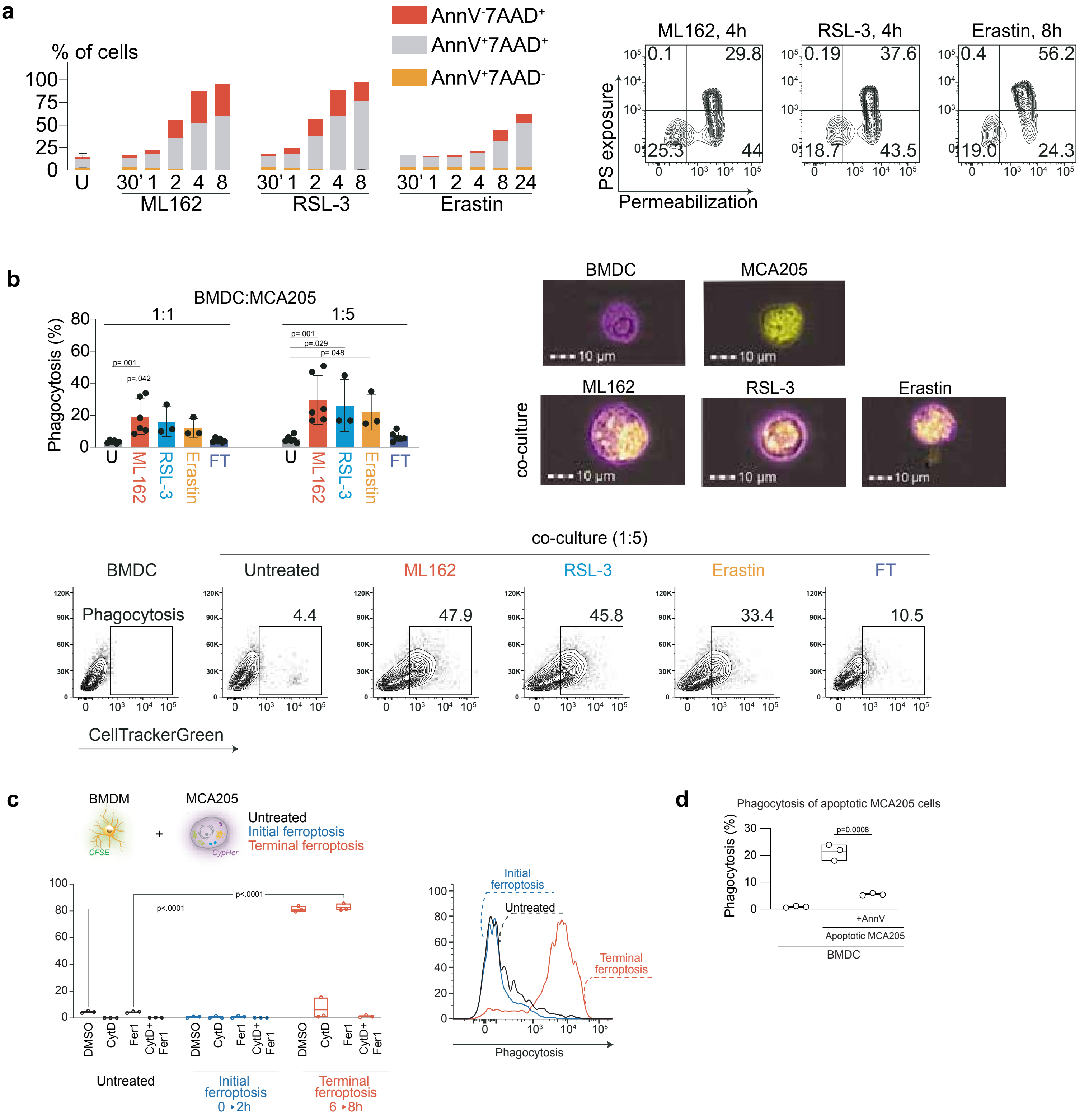

Supplementary Figure 6. Chemically-induced ferroptotic cells are engulfed by BMDC in phosphatidylserine-independent manner.

a. Flow cytometry analysis of Annexin V and 7-AAD staining of MCA205 cells stimulated with ML162 (0.5 µM), RSL-3 (0.5 µM) and Erastin (2.0 µM) at different time points. Data presented as mean from n=2 independent samples. Contour plots represent gating strategy. **b**. The % of phagocytic dendritic cells incubated with untreated, ferroptotic or necrotic cells labelled with CellTrackerGreen. Data comes from n=6 biological replicates for untreated, ML162-treated and FT necrotic cells and n=3 for RSL-3 and Erastin-treated MCA205 cells and is presented as mean ± SEM of % $CD11c^{+}CellTrackerGreen^{+}$. Data analyzed by one-way ANOVA with Dunnet's post-hoc test with p values representing comparison to BMDC incubated with live (untreated) MCA205 cells. Photographs represent the intracellular presence of the cargo. Contour graphs represent the gating strategy. **c.** The analysis of phagocytosis by macrophages at different stages of ferroptotic cell death. CypHer-labelled MCA205 cells were incubated with CFSE-labelled bone marrow derived macrophages for 2 hours. Afterwards the phagocytosis was determined by the detection of CypHer fluorescence in CFSE-stained BMDCs. Cytochalasin D and Fer1 were used as inhibitors of phagocytosis and lipid peroxidation respectively. Data comes from n=3 biologically independent replicates and is presented as floating bar with bounds representing the range and center showing the mean of the collected data. Two-way ANOVA with Dunnet's post-hoc test, P values show the comparison to the untreated condition with the same inhibitor. **d**. The analysis of the phagocytosis of apoptotic MCA205 cells pretreated with recombinant Annexin V. Data comes from n=3 biological replicates and is presented as floating bars with limits showing the range and the center showing the mean of the obtained values. Two-sided t-test between targets (MCA205) treated or untreated with Annexin V.

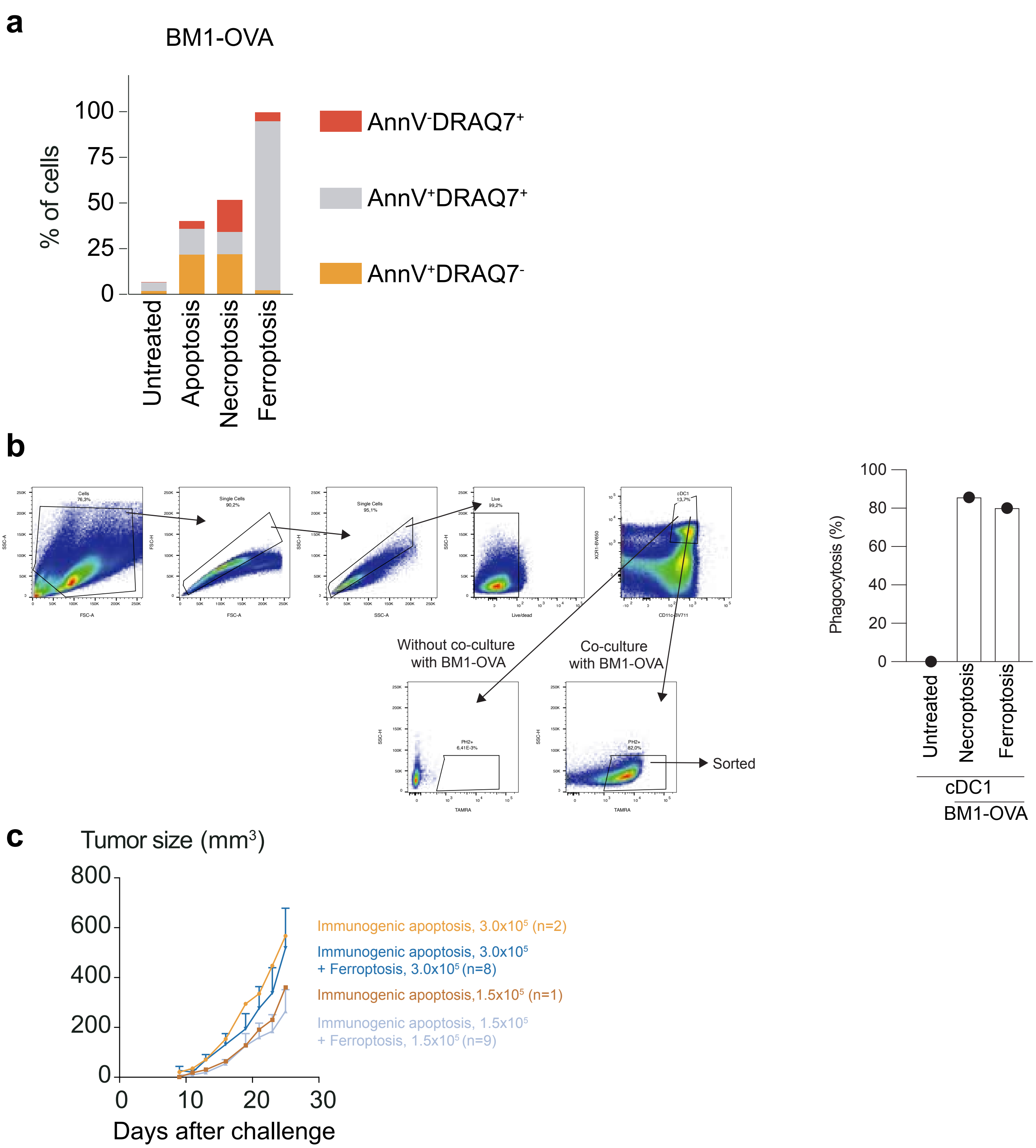

Supplementary Figure 7. The analysis of cells used in prophylactic and therapeutic vaccination model.
a. Cell death of BM1-OVA cells injected as vaccines in prophylactic vaccination model. **b**. Gating strategy and the level of phagocytosis of ferroptotic and necroptotic BM1-OVA cells by cDC1 in therapeutic vaccination model. **c.** Tumor size on the challenge site after the vaccination with mixtures of apoptotic and ferroptotic cells. Only data from the animals that succumbed to challenge is presented mean ± SEM.
