# Supplementary material for: Cancer cells dying from ferroptosis impede dendritic cell-mediated anti-tumor immunity
Source: Nat Commun. 2022 Jun 27;13:3676. doi: 10.1038/s41467-022-31218-2 (PMC9237053; doi:10.1038/s41467-022-31218-2)
Supplement: Supplementary file 3 — Description of Additional Supplementary Files [file 41467_2022_31218_MOESM3_ESM.pdf]

Description of Additional Supplementary Files

Supplementary Video 1
iGPX4KD MCA205 cells were seeded in the presence of doxycycline (1 µg/ml) and Ferrostatin-1 (0.5 µM) and SytoxGreen (1 µM) as a cell membrane permeabilization marker. After 48 h, cells were washed with fresh medium without Ferrostatin 1 and doxycycline. Subsequent culture led to synchronized cell death.

Supplementary Video 2
iGPX4KD MCA205 cells were seeded in the presence of doxycycline (1 µg/ml) and Ferrostatin-1 (0.5 µM) and SytoxGreen (1 µM) as a cell membrane permeabilization marker. After 48 h, cells were washed with fresh medium with Ferrostatin 1. Subsequent culture rescues cell death.
