# Supplementary material for: Cancer cells dying from ferroptosis impede dendritic cell-mediated anti-tumor immunity
Source: Nat Commun. 2022 Jun 27;13:3676. doi: 10.1038/s41467-022-31218-2 (PMC9237053; doi:10.1038/s41467-022-31218-2)
Supplement: Supplementary file 6 — Reporting Summary [file 41467_2022_31218_MOESM6_ESM.pdf]

nature portfolio

Corresponding author(s): Prof. Dr. Peter Vandenabeele

Last updated by author(s): 05/04/2022

Reporting Summary

Nature Portfolio wishes to improve the reproducibility of the work that we publish. This form provides structure for consistency and transparency in reporting. For further information on Nature Portfolio policies, see our Editorial Policies and the Editorial Policy Checklist.

Statistics

For all statistical analyses, confirm that the following items are present in the figure legend, table legend, main text, or Methods section.

n/a	Confirmed	
☐	☒	The exact sample size (n) for each experimental group/condition, given as a discrete number and unit of measurement
☐	☒	A statement on whether measurements were taken from distinct samples or whether the same sample was measured repeatedly
☐	☒	The statistical test(s) used AND whether they are one- or two-sided<br>*Only common tests should be described solely by name; describe more complex techniques in the Methods section.*
☒	☐	A description of all covariates tested
☐	☒	A description of any assumptions or corrections, such as tests of normality and adjustment for multiple comparisons
☐	☒	A full description of the statistical parameters including central tendency (e.g. means) or other basic estimates (e.g. regression coefficient) AND variation (e.g. standard deviation) or associated estimates of uncertainty (e.g. confidence intervals)
☐	☒	For null hypothesis testing, the test statistic (e.g. F, t, r) with confidence intervals, effect sizes, degrees of freedom and P value noted<br>*Give P values as exact values whenever suitable.*
☒	☐	For Bayesian analysis, information on the choice of priors and Markov chain Monte Carlo settings
☒	☐	For hierarchical and complex designs, identification of the appropriate level for tests and full reporting of outcomes
☒	☐	Estimates of effect sizes (e.g. Cohen's d, Pearson's r), indicating how they were calculated

Our web collection on statistics for biologists contains articles on many of the points above.

Software and code

Policy information about availability of computer code

Data collection	For flow cytometry experiments, data was collected using FACSDIVA 8.0 software. For Image Stream experiments, data was collected using INSPIRE 6.0 software.
Data analysis	Data was analyzed using FlowJo v10, Graphpad Prism v9.0, Microsoft Excel v16.5. Pictures were extracted using IDEAS 6.2 software (for ImageStream experiments) and Fiji v1.53 built on ImageJ 2.1.0 (for live cell imaging experiment). RNA sequencing was performed using R packages Rsubread. Pathway analysis was performed using GO and GSEA (gene sets from MSigDB were used including HALLMARKS, KEGG and gene ontology terms, BIO PROCESS and MOLECULAR FXN).

For manuscripts utilizing custom algorithms or software that are central to the research but not yet described in published literature, software must be made available to editors and reviewers. We strongly encourage code deposition in a community repository (e.g. GitHub). See the Nature Portfolio guidelines for submitting code & software for further information.

Data

Policy information about availability of data

All manuscripts must include a data availability statement. This statement should provide the following information, where applicable:

- Accession codes, unique identifiers, or web links for publicly available datasets
- A description of any restrictions on data availability
- For clinical datasets or third party data, please ensure that the statement adheres to our policy

All data generated or analysed during this study are included in this published article (and its supplementary information files).

Field-specific reporting

Please select the one below that is the best fit for your research. If you are not sure, read the appropriate sections before making your selection.

☒ Life sciences ☐ Behavioural & social sciences ☐ Ecological, evolutionary & environmental sciences

For a reference copy of the document with all sections, see nature.com/documents/nr-reporting-summary-flat.pdf

Life sciences study design

All studies must disclose on these points even when the disclosure is negative.

Sample size	For the in vivo experiments, sample size estimation was done by using G power 3.1.9.7. (alpha=0.05, beta=0.1) or based on the previous experience maintaining the balance between reaching the statistical significance and minimizing the number of used animals, and following already published papers in the context of immunogenic cell death (PMID:27050509, 31900335, 33230108, 31324798, 17187072). For the in vitro studies, the minimum of 3 independent measurements were used allowing to perform statistical tests and complying with previously published work (PMID: 31900335, 23852373).
Data exclusions	In the therapeutic vaccination model, two animals growing tumors intramuscularly were excluded and were not included in the data analysis.
Replication	The in vivo experiments involving therapeutic vaccination was performed once and was blinded. The rest of the in vivo experiments was performed at least twice. The in vitro experiments were performed at least three times, unless stated otherwise (in that case only representative experiment is shown). All of the experiments were reproducible provided the quality of the experiments (e.g. the expected result of the positive and negative controls) was ensured.
Randomization	Allocation of samples and organisms into experimental groups was random.
Blinding	For cell-based assays, investigators were not blinded in group allocation during data collection or analysis, because the researchers needed to keep track of the experiments and the analysis was performed by the same personnel. For the in vivo experiments, researcher measuring the tumor size was blinded.

Reporting for specific materials, systems and methods

We require information from authors about some types of materials, experimental systems and methods used in many studies. Here, indicate whether each material, system or method listed is relevant to your study. If you are not sure if a list item applies to your research, read the appropriate section before selecting a response.

Materials & experimental systems

n/a	Involved in the study	
☐	☒	Antibodies
☐	☒	Eukaryotic cell lines
☒	☐	Palaeontology and archaeology
☐	☒	Animals and other organisms
☒	☐	Human research participants
☒	☐	Clinical data
☒	☐	Dual use research of concern

Methods

n/a	Involved in the study	
☒	☐	ChIP-seq
☐	☒	Flow cytometry
☒	☐	MRI-based neuroimaging

Antibodies

Antibodies used	CD11c-APC (#117309, N418, BioLegend,), Fc Block antibody (553142, BD Pharmingen), MHCII-APC/eFluor780 (#47-5321-82, clone M5/114.15.2, eBioscience), CD86-Pe/Cy7 (#105116, clone PO3, BioLegend), CD80-Pe/Cy5 (#104712, clone 16-10A1, BioLegend), CD40-APC (#558695, clone 3/23, BD Pharmingen) CD274-PE (#12-5982-82, clone MIH-5, eBioscience), CD11c-BV650 (#117339, clone N418, BioLegend), calreticulin (#ab2907, Abcam), isotype control (#PA5-23094, ThermoFisher), secondary antibody for calreticulin detection goat anti-rabbit IgG (#35553, Thermo Fisher Scientific), XCR1-BV650 (#148220, clone ZET, BioLegend), CD11c-BV711 CD11c (#563048, clone HL3, BD Pharmingen), p-ikba (Ser32/36) (9246, Cell Signaling Technology), ikba (9242, Cell Signaling Technology), GPX4 (#ab125066, clone EPNCIR144, Abcam) , tubulin-HRP (#ab21058, Abcam), actin (#69100, clone C4, MP), CD3 (#56-0032-82, clone 17A2, ThermoFisher), CD8 (#25-0081-82, clone 53-6.7, ThermoFisher), isotype control (#PA5-33204, ThermoFisher) for calreticulin measurements.
Validation	All antibodies are from commercial sources and their validation data are available on the manufacturer's website:<br>CD11c-APC (#117309, N418, BioLegend,), https://www.biolegend.com/en-us/search-results/apc-anti-mouse-cd11c-antibody-1813?Clone=N418<br>Fc Block antibody (553142, BD Pharmingen), https://www.bdbiosciences.com/en-eu/products/reagents/flow-cytometry-reagents/research-reagents/single-color-antibodies-ruo/purified-rat-anti-mouse-cd16-cd32-mouse-bd-fc-block.553142<br>MHCII-APC/eFluor780 (#47-5321-82, clone M5/114.15.2, eBioscience), https://www.thermofisher.com/antibody/product/MHCClass-

II-I-A-I-E-Antibody-clone-M5-114-15-2-Monoclonal/47-5321-82
CD86-Pe/Cy7 (#105116, clone PO3, BioLegend), https://www.biolegend.com/en-us/search-results/pe-cyanine7-anti-mouse-cd86-antibody-3045?Clone=PO3
CD80-Pe/Cy5 (#104712, clone 16-10A1, BioLegend), https://www.biolegend.com/en-us/products/pe-cyanine5-anti-mouse-cd80-antibody-2339?Clone=16-10A1
CD40-APC (#558695, clone 3/23, BD Pharmingen), https://www.bdbiosciences.com/en-be/products/reagents/flow-cytometryreagents/
research-reagents/single-color-antibodies-ruo/apc-rat-anti-mouse-cd40.558695
CD274-PE (#12-5982-82, clone MIH-5, eBioscience), https://www.thermofisher.com/antibody/product/CD274-PD-L1-B7-H1-Antibody-clone-MIH5-Monoclonal/12-5982-82
CD11c-BV650 (#117339, clone N418, BioLegend), https://www.biolegend.com/en-us/products/brilliant-violet-650-anti-mouse-cd11cantibody-
8840?Clone=N418
calreticulin (#ab2907, Abcam), https://www.abcam.com/calreticulin-antibody-er-marker-ab2907.html
isotype control (#PA5-33204, ThermoFisher), https://www.thermofisher.com/antibody/product/Mouse-IgG1-Isotype-Control/PA5-33204
secondary antibody for calreticulin detection goat anti-rabbit IgG (#35553, Thermo Fisher Scientific), https://www.thermofisher.com/antibody/product/Goat-anti-Rabbit-IgG-H-L-Cross-Adsorbed-Secondary-Antibody-Polyclonal/35553
XCR1-BV650 (#148220, clone ZET, BioLegend), https://www.biolegend.com/en-gb/products/brilliant-violet-650-anti-mouse-rat-xcr1-antibody-12421?Clone=ZET
CD11c-BV711 (#563048, clone HL3, BD Pharmingen),https://www.bdbiosciences.com/en-be/products/reagents/flow-cytometryreagents/
research-reagents/single-color-antibodies-ruo/bv711-hamster-anti-mouse-cd11c.563048
p-ikba (Ser32/36) (9246, Cell Signaling Technology), https://www.cellsignal.com/product/productDetail.jsp?productId=9246
ikba (9242, Cell Signaling Technology), https://www.cellsignal.com/products/primary-antibodies/ikba-antibody/9242
GPX4 (#ab125066, clone EPNCIR144, Abcam), https://www.abcam.com/glutathione-peroxidase-4-antibody-epncir144-ab125066.html?productWallTab=ShowAll
tubulin-HRP (#ab21058, Abcam), https://www.abcam.com/hrp-beta-tubulin-antibody-loading-control-ab21058.html?productWallTab=ShowAll,
actin (#69100, clone C4, MP), https://www.mpbio.com/eu/08691001-mouse-anti-actin-monoclonal-clone-c4-cf
CD3-AF700 (#56-0032-82, clone 17A2, ThermoFisher), https://www.thermofisher.com/antibody/product/CD3-Antibody-clone-17A2-Monoclonal/56-0032-82
CD8 (#25-0081-82, clone 53-6.7, ThermoFisher), https://www.thermofisher.com/antibody/product/CD8a-Antibody-clone-53-6-7-Monoclonal/25-0081-82

Eukaryotic cell lines

Policy information about cell lines

Cell line source(s)	MCA205 cell line was a gift from prof. Guido Kroemer laboratory and previously described in PMID:25050214 and had been purchased from ATCC, Jurkat cells were purchased from ATCC, B16-OVA were previously described in PMID:29158474 and come from the VIB cell bank, BM1-OVA cell line was a gift from Prof. Caetano Reis e Sousa from the Francis Crick Institute.
Authentication	The cell lines were not authenticated.
Mycoplasma contamination	All of the cell cultures were tested regularly for the Mycoplasma presence. All of the tests were negative for Mycoplasma.
Commonly misidentified lines (See ICLAC register)	None of the used cell lines are listed in ICLAC register as commonly misidentified.

Animals and other organisms

Policy information about studies involving animals; ARRIVE guidelines recommended for reporting animal research

Laboratory animals	6-8 weeks female C57BL/6J purchased from Janvier Labs.
Wild animals	No wild animals were used.
Field-collected samples	No field collected samples were used.
Ethics oversight	All experiments were approved by the animal ethics committee of Ghent University and conducted according to institutional, national and European animal regulations.

Note that full information on the approval of the study protocol must also be provided in the manuscript.

Flow Cytometry

Plots

Confirm that:

☒ The axis labels state the marker and fluorochrome used (e.g. CD4-FITC).

☒ The axis scales are clearly visible. Include numbers along axes only for bottom left plot of group (a 'group' is an analysis of identical markers).

☒ All plots are contour plots with outliers or pseudocolor plots.

☒ A numerical value for number of cells or percentage (with statistics) is provided.

Methodology

Sample preparation	For the antibody staining, cells were collected and washed twice with ice-cold FACS Buffer (3% fetal calf serum in PBS), stained with the antibodies for 30 minutes in FACS buffer in 4oC in the dark, washed twice in ice-cold FACS buffer and resuspended in FACS buffer before measurements
Instrument	LSRII, LSR Symphony were used for flow cytometry experiments. Aria III was used for sorting.
Software	The data was collected using DIVA 8.0 software and further analyzed by FlowJo v10.
Cell population abundance	For the sorting of the BMDC engulfing the ferroptotic Jurkat cells, the cells were sorted based on the CellTraceViolet and TAMRA positivity (TAMRA was used to stain target cells). About 25-30% cells were positive. Around 1mln cells were sorted for further RNAseq analysis.<br>The population of cDC1 engulfing ferroptotic or necroptotic cells, CD11c+XCR1+TAMRA+ cells were sorted (about 80% of the population of cDC1). 4.5mln cells was collected for each of the condition.
Gating strategy	In all of the flow cytometry experiments, the initial gating was performed in a similar manner, viz. 1. FSC-A vs SSC-A: exclusion of the debris based on the size of the particles. 2. FSC-A vs FSC-H and SSC-A and SSC-H: exclusion of cell doublets based on the proportional increase of the area and height of the peak in both forward and site scatter. 3. Exclusion of dead cells by the permeability marker (SytoxBlue/7-AAD/DRAQ7). Further gating strategy is presented in the Extended Data.

☒ Tick this box to confirm that a figure exemplifying the gating strategy is provided in the Supplementary Information.
